# A simple rule for quadrupedal gait generation determined by leg loading feedback: a modeling study

**DOI:** 10.1038/srep08169

**Published:** 2015-02-02

**Authors:** Yasuhiro Fukuoka, Yasushi Habu, Takahiro Fukui

**Affiliations:** 1 Department of Intelligent Engineering, College of Engineering, Ibaraki University, 4-12-1 Nakanarusawa-cho, Hitachi-shi

## Abstract

We discovered a specific rule for generating typical quadrupedal gaits (the order of the movement of four legs) through a simulated quadrupedal locomotion, in which unprogrammed gaits (diagonal/lateral sequence walks, left/right-lead canters, and left/right-lead transverse gallops) spontaneously emerged because of leg loading feedbacks to the CPGs hard-wired to produce a default trot. Additionally, all gaits transitioned according to speed, as seen in animals. We have therefore hypothesized that various gaits derive from a trot because of posture control through leg loading feedback. The body tilt on the two support legs of each diagonal pair during trotting was classified into three types (level, tilted up, or tilted down) according to speed. The load difference between the two legs led to the phase difference between their CPGs via the loading feedbacks, resulting in nine gaits (3^2^: three tilts to the power of two diagonal pairs) including the aforementioned.

It is well known that quadrupeds select their gaits—i.e., the patterns of movement of their limbs (e.g., walking, trotting, cantering, galloping) according to speed^1^. Attracted to this phenomenon, a number of researchers have energetically discussed the possible ways gaits may be switched and the factors that cause them in a variety of fields (e.g., physiology, physics, mathematics). The group of spinal neurons that produces the motor pattern of each leg of an animal is called the central pattern generator (CPG), and the interlimb coordination with a particular type of gait requires different sets of interactions among the CPGs or changes in the connection strength^2^,^3^. It has been reported that the switching of the gaits is caused by energetic (to minimize energetic cost)^4^, durability (to protect from overload)^5^,^6^, biomechanical^7^, environmental and morphometric^8^, as well as mathematical^9^ factors. Thus, in terms of the gait transition, most researchers have concluded in general that given factors are variously responsible for different types of switching between existing gaits. However, few studies in these fields have focused on the principle of the generation of each gait itself, i.e., why an animal swings its legs in such an order in each gait.

Locomotion emerges from the biomechanics between the body and the environment, as well as the nervous system^10^. Modeling studies, such as those involving computer simulations and robots, are suitable for understanding such dynamical interactions. In addition, since a modeled animal can consist of only limited elements, unlike a test animal in physiological studies, its use allows a clear understanding of how each element contributes to the whole system. Specifically, Pearson et al.^11^ asserted that simulations enable the examination of the contribution of specific sensory and motor signals to overall function. Accordingly, in recent years, some modeling studies have attempted to reveal the gait generation/transition phenomenon with computer simulation models^12^,^13^,^14^,^15^ or robots^16^,^17^,^18^,^19^,^20^,^21^,^22^,^23^. All of the studies discussed the gait generation and/or transition mechanism of walking, except for one study on the gait generation of running^13^. However, none of them discovered a principle that consistently accounts for the gait generation from walking to running. Following the theory of motion generation proposed in the studies of physiology and biomechanics, we contributed to this literature by building simple quadruped robots^24^,^25^ and a simulation model^26^ and observed the effects of the dynamics of the locomotion in their gaits to search for a rule behind the generation of each gait. As a result, we believe that gait variation relates to posture control based on the following background.

Approximately a decade ago, we developed a biologically-inspired quadruped robot with the aim of walking over uneven terrain^24^. Its single CPG model produced the rhythm of each leg, the lateral neighboring CPG models were mutually and inhibitorily coupled, and the CPG network was always hard-wired. Therefore, the robot was able to move on flat terrain in a trot, which is a gait in which the diagonal pairs of legs move in phase, yet the two pairs together move out of phase. Since it has been reported that the sensory feedbacks to CPGs play important roles in synchronizing the oscillations of the CPGs with those of body parts^2^,^27^,^28^, we also applied the feedback of the body tilt around the roll axis to the CPG models, based on the vestibulospinal reflexes^29^ for postural control. This extended the stance duration of each leg on the side towards which the body tilted in the lateral plane and shortened that of each leg on the opposite side. As a result, since the phase of the CPG for each leg was autonomously fine-tuned according to the irregular body oscillation over uneven terrain, the robot was capable of dynamic robust walking over uneven terrain in a free gait, in which each leg phase irregularly deviates from trotting. In addition, we found that the vestibular modulation contributed to the emergence of an unprogrammed lateral sequence walk at low speeds on flat terrain. Since the cyclic duration of walking becomes longer at low speeds, the body regularly sways during the double support phase of the trot. We therefore inputted this body oscillation into each CPG through the vestibular feedback; as a result, each leg phase regularly deviated from trotting and the lateral sequence walk spontaneously emerged. On the other hand, since the body hardly swayed during trotting at medium speeds because of the short cyclic duration of walking, the vestibular feedbacks were not stimulated and the trot dominated by the CPG network was maintained.

In addition, when we applied the vestibular modulation to the body oscillation around the pitch axis during running on flat terrain, the two legs that the model tilted towards led to the stance phase according to its regular large body oscillation of backwards and forwards, and the model therefore achieved a safe bounding^25^.

Thus, since the posture control through the vestibular modulation had influenced the generation of the free gait over irregular terrain, and the lateral sequence walk and the bounding on flat terrain, we supposed that there was some relation between gaits and posture control.

In order to reveal the intrinsic principle of gait generation, we conducted a simulation study for which we built a simple planar quadruped model with the same control method in the sagittal plane^26^. As a result, we observed that not only a lateral sequence walk but also a transverse gallop spontaneously emerged from a trot as a basic gait because of the model's body oscillation in the sagittal plane. Moreover, we observed that the model's gaits autonomously transitioned from a walk at slow speeds to a trot at medium speeds, and then to a transverse gallop at high speeds following its acceleration, and vice versa following its deceleration. We therefore hypothesized that the typical gaits of quadrupedal locomotion are generated in relation to the posture control on the basis of the vestibulospinal reflexes.

Despite this hypothesis, however, none of the physiological studies have in fact revealed the existence of the feedback of vestibular information to the CPG. Moreover, in terms of postural control for terrestrial animals, Deliagina et al.^30^ reported that information on the stabilization of the head and body orientation is delivered by the sensory inputs of various modalities (vestibular, visual, and somatosensory), and that neither vestibular nor visual feedback is essential for the postural stabilization of terrestrial animals; somatosensory input is rather the main source of information. Accordingly, the posture of the terrestrial locomotion should be mainly controlled through the somatosensory feedback. Duysens^31^ mentioned that locomotion is regulated through feedback from various load receptors to central circuits involved in the generation of rhythmic locomotor output, and therefore, activity in antigravity muscles is promoted while the onset of the next flexion is delayed as long as the limb is loaded. More specifically, Pearson^32^ suggested the existence of a negative sensory feedback from load-sensitive receptors in the ankle extensor muscles to the flexor half-center in the CPG (Fig. 1), which prolongs the stance duration while the leg is loaded, through some animal walking experiments in variable environments (e.g., involving adaptation to treadmill speed^33^ or loss of ground support^34^). Computer simulation studies^35^,^36^ have demonstrated that posture control through leg loading feedbacks to CPGs is useful for walking over a step and climbing a slope. Based on these findings, we decided to use a leg loading feedback mechanism to each CPG proposed by Pearson (Fig. 1) for the posture control of our new quadruped model instead of vestibular feedback. The new model has the same mechanism and neural system as our previous model^26^, except for the inclusion of the leg loading feedback mechanism.

Its hard-wired CPG network, in which the lateral neighboring CPGs were mutually inhibited, originally produced a trot gait, such as in our previous model. However, in our new model, since the relation between the legs' loads varied according to the body tilt that changed based on speed, more types of gait (e.g., diagonal/lateral sequence walks at only low speeds, left/right-lead canters at only moderate high speeds, left/right-lead transverse gallops at only very high speeds, as well as a trot at medium speeds, during which the body hardly oscillates) spontaneously emerged. The gaits also autonomously transitioned from one to the other (e.g., from the lateral sequence walk to the trot to the left-lead canter to the left-lead gallop and vice versa) while the speed was continuously changed. Although all of the gaits were unprogrammed, except for the trot that was derived from the hard-wired CPG network, all were gaits that are naturally observed in animals. Based on the results, we revealed a rule that accounts consistently for gait generation from walking to running. We will detail this discovery in “Discussion” and hypothesize that the generation of each gait is attributed to a primitive neuro-mechanical factor that is related to posture control through leg loading feedback.

## Results

### Basic locomotion without leg loading feedback

Firstly, we will describe our basic quadruped model without the leg loading feedback. Based on the general conclusion that “A cat has two types of coordinating influences between ipsilateral legs and between contralateral legs, not direct connections between diagonal legs”^31^, its lateral neighboring CPG models are mutually and inhibitorily coupled (Fig. 7(a)), as in our previous model^26^; therefore, the hard-wired CPG network originally produces a trot. The values of all of the parameters used in our quadruped model are set to constant at a constant speed, and were empirically determined so that the model could locomote safely. To change the speed, we only adjust several of the control parameters of each leg, called the “speed parameters (detailed in Section 4 of Supplementary Methods 1)”, in the same way in all of the legs, in order to determine the cycle and magnitude of the swinging of each leg; therefore, the adjustment of the speed parameters does not affect the interlimb coordination. It should be noted that the coupling parameters among the CPGs are also always fixed. All the parameters are empirically determined so that the model can safely walk.

Here, we allowed the simulated basic quadruped model without the leg loading feedback to locomote at an approximately constant acceleration (0.14 m/s^2^) and deceleration (−0.14 m/s^2^) in the sagittal plane, as shown in Fig. 2. We observed that the model was always safely trotting, during which the footfalls were anti-phase between the neighboring legs and in-phase between the diagonal legs, irrespective of changes in the speed. This demonstrated that the locomotion was dominated by the trot produced by the hard-wired CPG network. We did not observe other gaits even though we performed the locomotion with a variety of acceleration and deceleration. It should be noted in Fig. 2 that even in all of the trotting, the body oscillation was large at low (e.g., 10–11 s, 46–47 s) and high (e.g., 28–29 s) speeds while small at medium speeds (e.g., 18–19 s, 38–39 s). This was because of the extensively long support phase at low speeds and the non-restraint during the flight phase at high speeds. At medium speeds, the support phase, along with the walking cyclic duration, was shorter than at slow speeds, and the flight phase was either non-existent or momentarily existent; therefore, the body hardly oscillated.

### Gait generation

**Figure 1:**
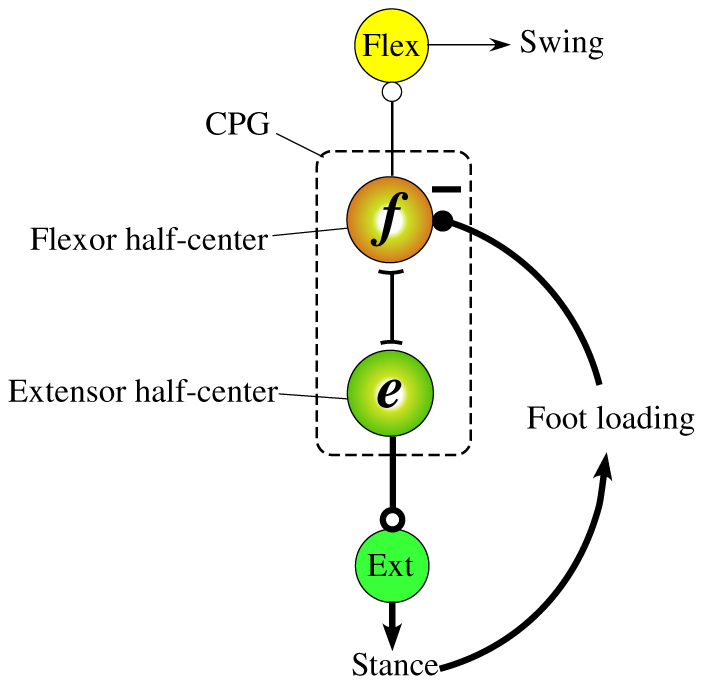
Modified figure from Fig. 1 of Pearson's work^32^. The CPG of each leg consists of a flexor half-center to activate the swing phase and an extensor half-center to activate the stance phase. The half-centers mutually inhibit one another. The load sensitive receptors in the ankle extensor muscles inhibit the flexor half-center. While the leg is loaded, the extensor half-center is excited because of the inhibition of the flexor half-center, which results in prolongation of stance duration and prevention of initiation of the swing phase. The timing of the stance-to-swing is controlled by leg loading feedback.

Next, we applied the leg loading feedback to the basic quadruped model. Specifically, the value detected by the load-sensitive receptor of each foot was multiplied by a negative constant gain, −*k*_2_ in Eq. (1), which was common in all of the legs and simply inputted into each flexor half-center, as shown in Fig. 1. Therefore, the stance duration was extended while the leg was loaded, resulting in an adjustment of the timing of the stance-to-swing transition. We allowed the quadruped model with the leg loading feedbacks to locomote at a variety of constant speeds by only adjusting the speed parameters mentioned in the previous section. As a result, a variety of unprogrammed, distinct, steady gaits observed in quadrupedal animals emerged. These simulations can be seen in the video “Supplementary Movie 1”. The data of the approximate four-step cycles of these emerged gaits, such as a lateral sequence walk (L-walk), a diagonal sequence walk (D-walk), a trot, a right-lead canter (R-canter), a left-lead canter (L-canter), a right-lead transverse gallop (RT-gallop), as well as a left-lead transverse gallop (LT-gallop), are shown in Fig. 3. In this figure, each term in parentheses represents the abbreviation of each gait that will be used in this paper. Flight phases appeared in the fast trot, the canters, and the gallops, which mean running. The main gaits (walking, trotting, cantering, and galloping) are subdivided by speed (e.g., walking at low speeds, trotting at medium speeds, and cantering and galloping at high speeds), and their subcategories (L or D in the walks, L or R in the canters, and T-gallops) are mainly divided by the posture or the final state of the previous gait, even at the same speed. We believe that these different gaits emerge because of the body oscillation variations according to the speed during trotting, as mentioned in the previous section. Even at other speeds, in addition to the ones in Fig. 3, the seven kinds of gaits above and the two kinds of unusual canters, which are similar to canters but are not seen in animals, were observed as steady gaits. This result validated the notion that these are not pinpointed gaits observed only at a particular speed. In addition, except for these nine kinds of gaits, no other steady gaits emerged.

### Gait transition

**Figure 2:**
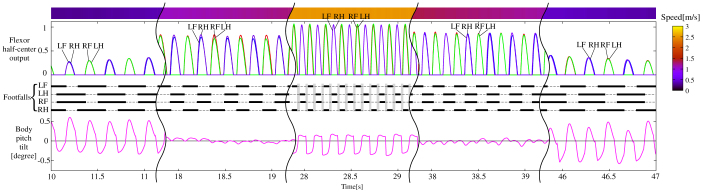
Simulation results for the quadruped model without the leg loading feedback at an approximately constant acceleration (0.14 m/s^2^) and deceleration (−0.14 m/s^2^). LF, LH, RF, and RH represent left foreleg, left hindleg, right foreleg, and right hindleg, respectively. The speed is shown as the top meter, whose speed level is denoted as the color meter on the right side. The top convex curves indicate the flexor half-center outputs for LF (blue), LH (red), RF (green), and RH (purple), which lead to the swing phase. The thick line segments on the thin dashed lines at the middle show each leg's footfall, where the light gray areas at high speeds (28–29 s) show the flight phases. The bottom plot shows the body tilt around the pitch axis (positive while tilting down). The trot is the locomotion modelled throughout the entire duration.

We found that when the speed changed over time, the gait transitioned among the gaits above, similarly to animals. Fig. 4 shows the data that resulted from allowing the quadruped model with the leg loading feedback to perform the same simulation as that in Fig. 2. Specifically, we used the same parameters, except for the leg loading feedback gain of each leg, throughout the entire durations of the two simulations in Figs. 2 and 4. The simulation can also be seen in the video “Supplementary Movie 2”. We observed that the gaits transitioned from an L-walk to a trot to an R-canter to an RT-gallop during acceleration and from an RT-gallop to R-canter to a trot to a D-walk during deceleration. In this simulation, the canters were only observed for short periods during acceleration and deceleration. We could observe them for longer durations at lower accelerations and decelerations. The canters only emerged over a small range of speeds, unlike the other gaits.

## Discussion

If two different feedback signals are inputted into the two CPGs of each diagonal pair of legs in phase during trotting, a different gait should emerge because the two CPGs would be prevented from being in phase. Based on this theory, we consider that the load difference between the two support legs of each diagonal pair, which is caused by the body tilt, leads to a particular gait from a trot, and suggest the following specific rule by which a variety of gaits can be generated.

**Figure 3:**
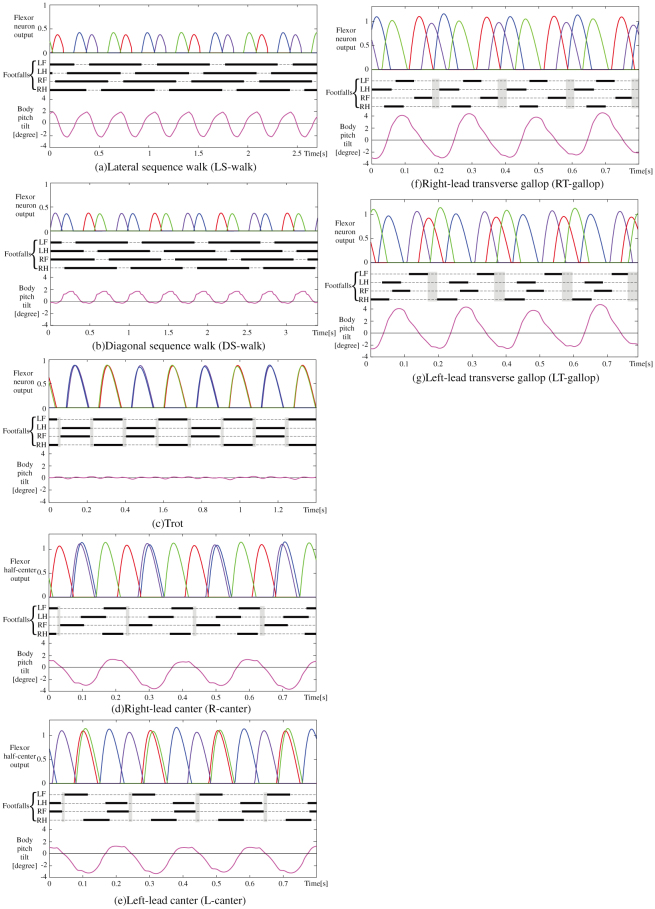
Simulation results for the quadruped model equipped with the leg loading feedback. Each graph shows the locomotion at each constant speed. The definitions of the plots in each graph are described in Fig. 2. Flight phases can be seen in the fast trot (c), canters (d, e), and gallops (f, g). In (a), the constant speed is 0.25 m/s. The flexor half-center outputs and the footfalls transition in the order LF-RH-RF-LH, which represents a lateral sequence walk (L-walk) mainly observed in the low-speed locomotion of animals excluding primates. In (b), the constant speed is 0.25 m/s. The flexor half-center outputs and the footfalls transition in the order LF-LH-RF-RH, which represents a diagonal sequence walk (D-walk) mainly observed in the low speed locomotion of primates. In (c), the constant speed is 1.1 m/s. The body oscillation is very small at medium speeds, resulting in a trot. In (d), the constant speed is 2.2 m/s. The flexor half-center outputs and the footfalls transition in the order (LF and RH in phase)-RF-LH, which represents a right-lead canter (R-canter) mainly observed as an intermediate between the trot and the gallop in animals. In(e), the constant speed is 2.2 m/s. The flexor half-center outputs and the footfalls transition in the order of LF-RH-(RF and LH in phase), which represents a left-lead canter (L-canter) also mainly observed as an intermediate between the trot and the gallop in animals. In (f), the constant speed is 2.5 m/s. The flexor half-center outputs and the footfalls transition in the order LF-RF-LH-RH, which represents a right-lead transverse gallop (RT-gallop) mainly observed in the high-speed running of animals. In (g), the constant speed is 2.5 m/s. The flexor half-center outputs and the footfalls transition in the order LF-RH-LH-RF, which represents a left-lead transverse gallop (LT-gallop) also mainly observed in the high-speed running of animals.

In this discussion, the left foreleg, left hindleg, right foreleg, and right hindleg are abbreviated to LF, LH, RF, and RH, respectively. Fig. 5(a) shows the diagram of the rhythmic activities of the flexor half-centers, which lead to the swing phases, of the four legs. All particular gaits start from the pattern of a trot, shown in green, observed at medium speeds. Following a change in speed (up or down), the phases of the two legs of each diagonal pair (1^st^ diagonal pair (RF and LH) and 2^nd^ diagonal pair (LF and RH)) begin to shift gradually with the same duration from the green curves in opposite directions (see the red or blue curves in each diagonal pair), while the origins of the two diagonal pairs are always kept as anti-phase (see the dashed lines). This divergence results in the generation of the different gaits from a trot. The emerged gaits are classified as typical types of gait according to the type of phase shift in each diagonal pair, which can be one of three types, represented by the green (no shift), red, and blue curves in each diagonal pair in Fig. 5(a). The three types of phase shift result from the three types of body tilts in the stance phases of the two legs of each diagonal pair during trotting (“tilted down”, “level”, or “tilted up” in Fig. 5(b)). For example, when the body is tilted down, such as on the left of Fig. 5(b), because the load of the foreleg is larger than that of the hindleg (compare the two pink arrows pointing up from the feet), the flexor half-center of the foreleg is more inhibited through the negative feedback from the load-sensitive receptor to the flexor half-center (Fig. 1). Therefore, the swing phase (flexor activation) of the foreleg becomes delayed with respect to that of the hindleg, as represented by the blue curves in Fig. 5(a). In contrast, when the body is tilted up, such as on the right of Fig. 5(b), the swing phase of the hindleg becomes delayed with respect to that of the foreleg, as seen in the red curves in Fig. 5(a). On the other hand, when the body is kept level as shown in the middle of Fig. 5(b), the leg load of the foreleg is equivalent to that of the hindleg and the flexor half-centers of the two legs are equally inhibited. Therefore, both swing phases of each diagonal pair are kept in-phase, as represented by the green curves in Fig. 5(a). Since each diagonal pair can be phase-shifted in three ways according to the three body tilts, nine ( = 3^2^) possible combinations of gaits exist altogether, as shown in Fig. 5(c). The order of the letters in the parentheses indicates the order of the legs upon entering the swing phase. For example, when the body is tilted up in the stance phases of the two legs in both diagonal pairs, as in the animals shown on the right side of Fig. 5(b), the four legs swing in the order RF-LH-LF-RH (red curves in Fig. 5(a)), which is the L-walk. This rule can account for the seven kinds of gaits that emerged in the simulation shown in Fig. 3, which are all included in Fig. 5(c). Two unusual canters are shown in the table that can only be observed in our model and not in any animal. Canters were not observable in our previous model^26^, which employed vestibular feedback, for the following reason. In our previous model, because of the vestibular modulation, the two legs that the model tilted toward in the sagittal plane led to the stance phase while the other legs led to the swing phase. This modulation worked even for the swing legs, unlike our current model. Thus, for example, if the previous model were in a tilted-up position, as shown in the top right of Fig. 5(b), a phase difference would occur even between the two swing legs of the 2^nd^ diagonal pair (i.e., LF would be led to the swing phase and RH would be led to the stance phase). After the two swing legs that deviate from each other subsequently touch the ground, the two support legs would tend not to become in phase and the posture would not become level as shown in the lower middle of Fig. 5(b), which would prevent the generation of the canters. Therefore, the current model with the leg load feedback should be more consistent with an animal.

Here, we will prove that the table in Fig. 5(c), which we created according to the above rules, is consistent with the simulation results.

- A trot As shown in Fig. 3(c), since there is little body oscillation at medium speeds because of the short cyclic duration and the short flight phase, the posture in each diagonal pair is always kept level. Therefore, the loads of the two legs of each diagonal pair are almost the same, and a phase difference between the two legs does not appear; as a result, a trot is maintained as shown in Fig. 5(c).
- Lateral/Diagonal sequence walks (L/D-walk) The body oscillates at low speeds of trotting because of the long support phase, as shown in Fig. 2. Fig. 6(a) shows a lateral sequence walk. The body was mainly tilted up in the latter of the stance phases of RF and LH of the 1^st^ diagonal pair (A); meanwhile, the leg load of LH (B) was larger and remained longer than that of RF (C). As a result, the flexor half-center of LH was inhibited more strongly, and therefore, LH changed to the swing phase after a delay from RF (D). The same applied for the 2nd diagonal pair (LF and RH)(A′–D′): the two legs changed to the swing legs in the order LF-RH (D′). This formed a lateral sequence walk (L-walk) as shown in Fig. 5(c)
- Fig. 6(b) shows a diagonal sequence walk. In contrast to the L-walk, the body was mainly tilted down in the latter of the stance phases of RF and LH of the 1^st^ diagonal pair (A); meanwhile, the leg load of RF (B) was larger and remained longer than that of LH (C). As a result, the flexor half-center of RF was inhibited more strongly, and therefore, RF changed to the swing phase after a delay from LH (D). The same applied for the 2^nd^ diagonal pair (LF and RH)(A′–D′): the two legs changed to the swing legs in the order RH-LF (D′). This formed a diagonal sequence walk (D-walk) as shown in Fig. 5(c).
- Although both the L-walk and the D-walk can emerge at the same range of speeds, which one emerged depended on whether the body was tilted up or down, respectively, in the latter of the stance phases of the two legs of each diagonal pair. In our simulation, the D-walk tended to occur when the center of the body oscillation inclined further forwards; otherwise, the L-walk would occur.
- Right/Left-lead canters (R/L-canters) and unusual canters The body oscillates at high speeds because of the unconstraint in the flight phase, as shown in Fig. 2. Fig. 6(c) shows a right-lead canter. Since the model was tilted up in the stance phases of the two legs (RF and LH) of the 1^st^ diagonal pair (A), the leg load of LH became larger than RF (B). The flexor half-center of LH was more strongly inhibited, and therefore, LH changed to the swing phase after a delay from RF (C). On the other hand, since the body was nearly level in the stance phases of the two legs (LF and RH) of the 2^nd^ diagonal pair (D), the pair's leg loads were close (E), and therefore, the two legs changed almost simultaneously to the swing phases (F). This formed a right-lead canter (R-canter). A left-lead canter (L-canter) formed when the pairs in the above explanation were switched. Unusual canters, which are not observable in any animal, appeared in the simulation when the model was tilted down instead of up during the R/L-canters. All of the results were consistent with the table in Fig. 5(c). Either a right- or left-lead canter would randomly emerge from a trot even at the same speeds with all of the same control parameters, which seemed to depend on the state at the moment of transitioning from a trot to each gait. Whether the R/L-canters or the unusual canters emerged depended on whether the body oscillation was leaning forward (unusual canters) or backward (R/L-canters) even at the same speeds, which was caused by the slight difference of the final posture of the previous gait. Canters are considered halfway gaits when the body oscillation leans either forward (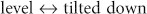) or backward (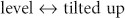) in the process of the transition between a trot and a gallop.
- Right/Left-lead transverse gallops (RT/LT-gallops) The body oscillates at high speeds because of the long flight phase, as shown in Fig. 2. Fig. 6(d) shows a right-lead transverse gallop (RT-gallop). Since the model was mainly tilted down in the stance phases of the two legs (LF and RH) of the 2^nd^ diagonal pair (A), the leg load of LF became larger than that of RH (B). The flexor half-center of LF was more strongly inhibited, and therefore, LF changed to the swing phase after a delay from RH (C). Conversely, since the model was mainly tilted up in the stance phases of the two legs (RF and LH) of the 1^st^ diagonal pair (D), the leg load of LH became larger than that of RF (E), and therefore, LH changed to the swing phase after a delay from RF (F). This formed a right-lead transverse gallop (RT-gallop), as shown in Fig. 5(c). In Fig. 6(d), the phase difference between RF and LH (F) of the 1^st^ diagonal pair became very large because of the significant difference between the loads of the two legs (E), and therefore, the flight phases were observable between their stance legs. A left-lead transverse gallop (LT-gallop) formed when the pairs in the above explanation were switched. The RT-gallop or the LT-gallop emerged either via the R-canter or the L-canter from a trot, respectively, or directly from a trot, mainly depending on the acceleration/deceleration.

A slight phase difference between the swing phases of the two legs of each diagonal pair while trotting was generated by the leg loading feedbacks, and the next stance phases started with the phase difference. In this way, the phase difference gradually extended from a trot, resulting in several steady gaits. Such a mutual entrainment between the neural system and the body was captured by the model's inclusion of leg loading feedbacks in its design.

**Figure 4:**
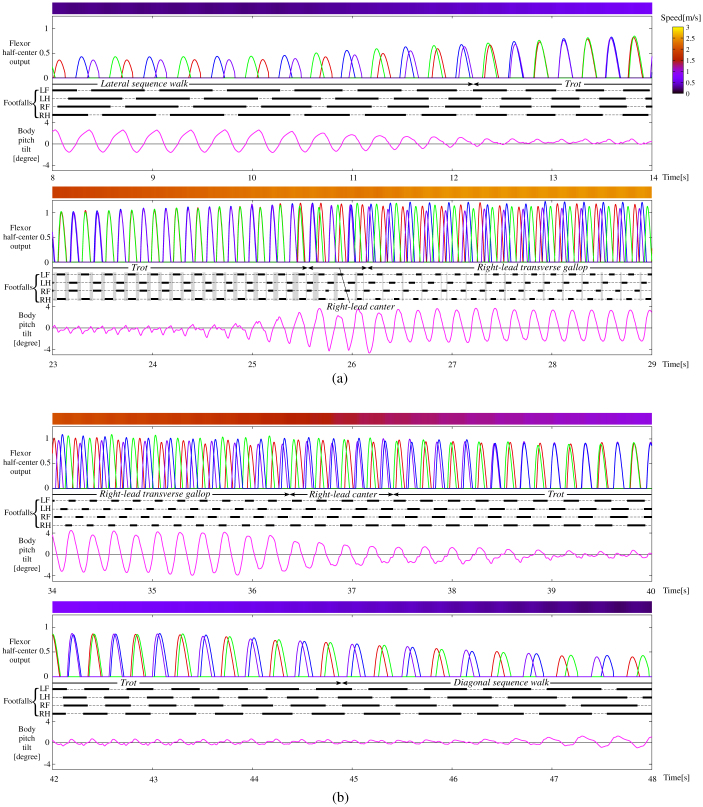
Simulation results for the quadruped model with the leg loading feedback during acceleration (a) and deceleration (b). We used the same parameters throughout the entire durations as in Figs. 2, except for the leg loading feedback gain of each leg. The definitions of the plots are the same as those in Fig. 2. It should be noted that the gaits shown in Fig. 3 transition according to speed as in a typical quadrupedal animal (specifically, from an L-walk to a trot to an R-canter to an RT-gallop in (a) and from an RT-gallop to R-canter to a trot to a D-walk in (b)).

**Figure 5:**
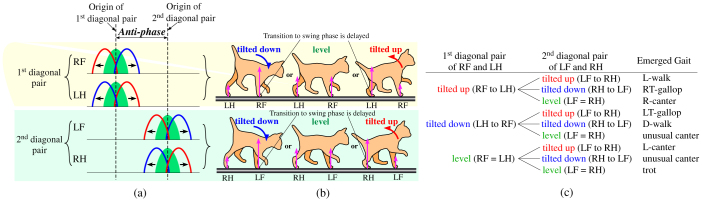
The rule of gait generation. LF, LH, RF, and RH represent left foreleg, left hindleg, right foreleg, and right hindleg, respectively; RF and LH belong to the 1^st^ diagonal pair while LF and RH belong to the 2^nd^ diagonal pair. (a) shows a diagram of the rhythmic activities of the flexor half-centers of the four legs. (b) shows the three types of postures (“tilted down”, “level”, or “tilted up”) in the stance phases of the two legs in phase of each diagonal pair during trotting. (c) shows the nine emerged gaits that result from the possible combination of two elements (two diagonal pairs) out of the set (tilted down, level, tilted up). When the body is tilted down or up, as shown in (b), the different loads are sensed between the two feet of each diagonal pair, as shown by the pink arrows. Each load's feedback is inhibitorily inputted into the flexor half-center of its leg, resulting in a phase difference between the two legs of each diagonal pair, as shown by the blue curves (tilted down) and red curves (tilted up) in (a). When the body is kept level, as shown in (b), the phase difference does not occur, as shown by the green curves in (a), because the loads are equivalent between the two support legs of each diagonal pair. The three types of tilts occur in each of the two diagonal pairs, resulting in the nine ( = 3^2^) possible combinations (particular gaits) shown in (c). See the main text for more details.

**Figure 6:**
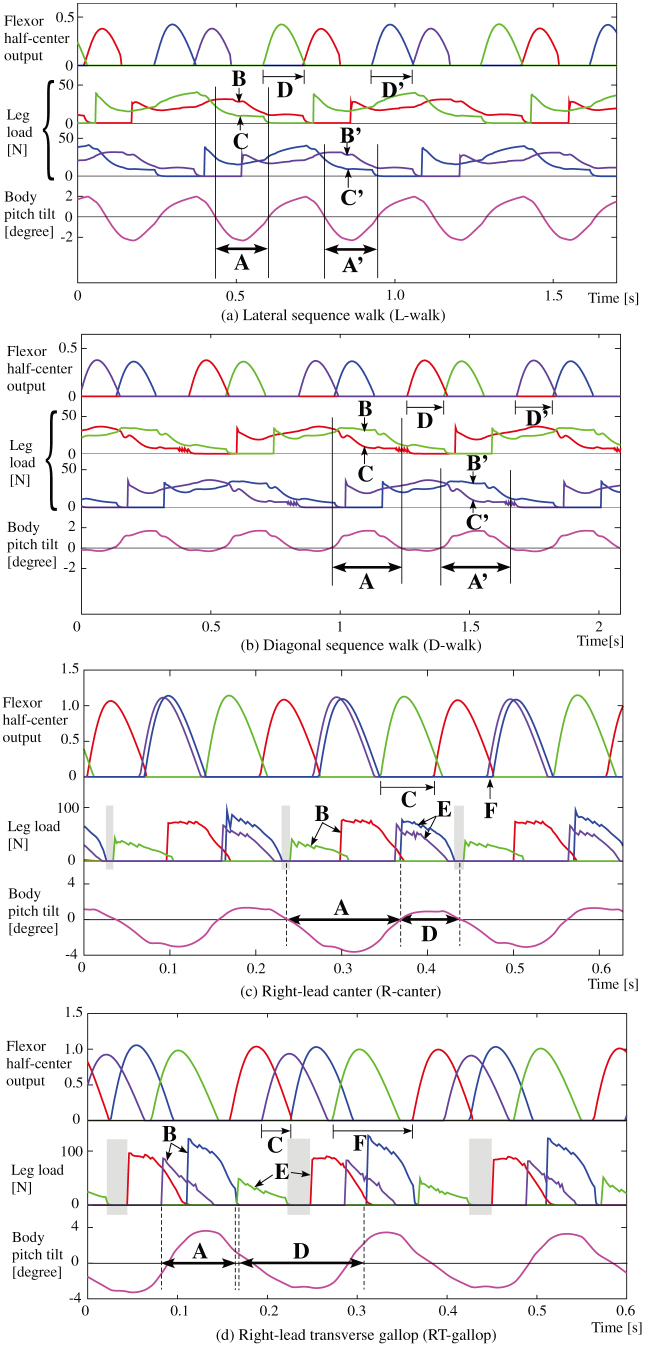
Locomotion data during an L-walk (a), a D-walk (b), an R-canter (c), and an RT-gallop (d). The top curves in each graph show the flexor half-center outputs of LF (blue), LH (red), RF (green), and RH (purple). The middle plots show the leg loads of LF (blue), LH (red), RF (green), and RH (purple), each of which is detected by the load-sensitive receptor. The light gray areas show the flight phases. Each bottom wave shows the body tilt (positive while tilted down).

Another important feature is that the nine gaits shown in Fig. 5(c) can be classified into gaits at three ranges of speed; in the simulation, we demonstrated that the walks, the trot, and the canters and gallops were only observed at low, medium, and high speeds, respectively: the same as in animals. We found that this was because the ratio between the frequency of each leg and that of the body was different. As shown in Fig. 2, where the model was trotting throughout the entire duration, that while little body oscillation was observed at medium speeds (e.g., 18–19 s, 38–39 s), the ratio between the frequency of each leg and that of the body was 1:2 at low speeds (e.g., 10–11 s, 46–47 s) and 1:1 at high speeds (e.g., 28–29 s). In biomechanical findings^37^, the ratio has tended to be 1:1 for running, and 1:2 for walking in bipedal locomotion. When the two legs of each diagonal pair in trotting are regarded as a single leg, the trot is similar to bipedal locomotion. At low speeds of trotting, since the body oscillates once for each diagonal pair swing, the same body tilt is present during the movement of both diagonal pairs. This feature at low-speed locomotion leads to the walks. It is evident in Fig. 5(c) that when the body was tilted up in both diagonal pairs, the gait became an L-walk, and when it was tilted down, the gait became a D-walk. On the other hand, at high speeds of trotting, since the half cycle of the body oscillation passed at one diagonal pair while the other half passed at the other diagonal pair, the body tilt was different between the two diagonal pairs, leading to L/R-canters, unusual canters or L/R-gallops, as shown in Fig. 5(c). The frequency rate between the body and the leg for each gait is evident in Figs. 3 and 4. Muybridge^38^ reported, for animal locomotion, that the movements of the head and neck (i.e. the body) exhibit a single peak per stride for a gallop, and double peaks per stride for a walk. Because of this particular feature, neither canters/gallops at low speeds nor walks at high speeds was observable in our simulation, as is the case with real animal locomotion.

Although the planar locomotion achieved by our simple quadruped model with limited elements is different from the actual locomotion of an animal, it is qualitatively very similar to the latter in many aspects. Therefore, we can at least suggest that the differences among an animal's leg loads caused by the posture variation in the sagittal plane may affect its gait generation. On the other hand, in terms of the gait transition, although our simulation demonstrated a gait transition that was similar to those of animals through approximately constant acceleration or deceleration, an animal tends to optimize a speed at the moment of each gait transition and chooses a speed within each gait in order to locomote efficiently. For example, it has been reported that a limit in musculoskeletal force triggers the gait transition from trotting to galloping^6^, and that a horse selects a small range of speeds that are energetically optimized within each gait^4^. Thus, if the speeds at which our quadruped model performs are optimized, it should be able to achieve a locomotion more similar to true animal locomotion. Finally, we hypothesize that the generation of each gait is attributed to a primitive neuro-mechanical factor related to posture control through the leg loading feedback, whereas the transitions among the gaits are highly and individually optimized.

## Methods

### A quadruped model in computer simulation

**Figure 7:**
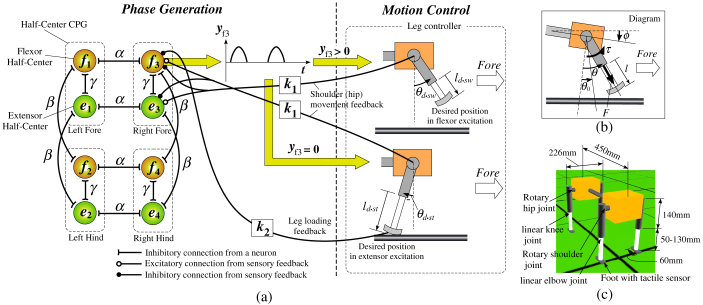
A configuration of our quadruped model. (a) shows the hard-wired CPG network that consists of the half-center CPG models that are mutually coupled and the motion controller of each leg (the controllers of the legs are excluded from the figure, except for the right foreleg). (b) shows a diagram of the model for the equations in Supplementary Methods 1. (c) shows a simple simulated planar quadruped model, which only has a rotary shoulder (hip) joint and linear elbow (knee) joint in each leg.

We utilized a dynamic simulator called Webots^39^, which has been used in many locomotion studies^20^,^26^,^36^,^40^,^41^. We built a simple quadruped model in Webots, as shown in Fig. 7(c). This model was the same as our previous one^26^, except that the new model was equipped with a tactile sensor on each foot to detect its leg loading. All of the four legs were identical. The model had a rigid body of 4.0 kg as its torso, to which each leg was attached through a rotary shoulder (hip) joint. Upper legs of 0.5 kg each and lower legs of 0.2 kg each were connected through a linear elbow (knee) joint capable of expansion and contraction. The joint worked as a passive compression spring in the stance phase, and the leg was retracted in the swing phase to avoid tripping. A small arched foot of 0.05 kg was fixed on the tip of each lower leg. The quadruped model with the eight joints weighed 7.0 kg in total. Although the elastic stick legs may seem to be far-fetched models, they were based on the spring loaded inverted pendulum (SLIP) model^42^,^43^, which is a simple single massless linear spring with a point-mass mounted on it. It has been mentioned in biomechanics that the locomotion of the SLIP model is very similar to the walking^37^ and the running^44^,^45^ locomotion of a variety of animals in terms of the trajectory of the center of mass, the vertical and fore-aft ground forces, and the mechanical energy. Therefore, the SLIP model has often been used for mathematical analyses of locomotion^46^,^47^,^48^. Although we could have built a realistic leg model that consisted of a number of rotary joints and muscle models, we decided to use the SLIP model, which is simple and mimics the features of an animal, in order to rule out complicating factors of leg parameters—e.g., number of joints, complexity of the muscle model, differences in length among the leg's segments, etc.—that could affect the success of gait generation. We placed the quadruped model between two sidewalls without friction to restrict its motion to the sagittal plane. Locomotion analyses in the sagittal plane have been generally implemented in the past^13^,^47^,^49^,^50^. The more specific effects of the body dynamics that occur in the sagittal plane are observable from planar locomotion.

### Half-center CPG model on each leg and the CPGs' network

It is a known fact that an animal produces a locomotion rhythm through the CPG. Neurophysiologists have discovered and paid particular attention to a half-center CPG composed of two groups (an extensor half-center and a flexor half-center) of spinal neurons that mutually inhibit one another^51^,^52^. The two half-centers send motor commands to the motoneurons of extensors and flexors, respectively. Although many numerical CPG models have been proposed^9^,^53^,^54^,^55^,^56^,^57^,^58^,^59^,^60^, Pearson et al.^11^ asserted, “To be useful for studying sensory feedback, the model of the central pattern generator does not need to include all the details of cellular and synaptic mechanisms, provided that it reacts to sensory input in accordance with experimental results”. Since we merely focused on the adaptation of each leg's phase caused by the leg loading feedback, we decided to use the simplest half-center CPG model proposed by Matsuoka^56^,^61^, as shown in Fig. 7(a). Matsuoka's model is a feasible mathematical half-center CPG model equipped with a small number of vital components, and many simulation models and robots with it have achieved adaptive locomotion^24^,^25^,^26^,^49^,^62^,^63^,^64^ and rhythmic arm movements^65^,^66^,^67^,^68^.

The CPG model for each leg is governed by several differential equations (see Section 1 of Supplementary Methods 1). In Fig. 7(a), the weights *α* and *β* determine the interlimb coordination and represent the connection strengths between the contralateral neurons and ipsilateral neurons, respectively, and the extensor and flexor half-centers on each leg mutually inhibit one another through the weight *γ*. Our model does not have connections between the diagonal legs, given the lack of evidence of their existence in animals^31^. The essential gait pattern is determined as a trot because of the mutual inhibitory connections through *α* and *β*. All of the parameter values of each CPG are the same for the four legs.

### Motion control of each leg

Although we control each leg according to the output from its half-center CPG, we only depend on its phase information and prepare a simple motor controller to move each leg because the moderately precise position control of the foothold in the swing phase is required for the stable locomotion while the elastic motion similar to the SLIP model is required in the stance phase. Therefore, first of all, as shown in the “Phase Generation” side of Fig. 7(a), the leg is led to the swing phase during the flexor half-center excitation (*y_fi_* > 0), otherwise (*y_fi_* = 0), it is in the stance phase; following each phase, each leg is controlled through the proportional derivative (PD) controller, as shown in the “Motion Control” side. During the swing phase, the leg is retracted by a target length (*l_d_*_·*sw*_) to avoid tripping and is swung forward to a target angle of the shoulder (hip) joint (*θ_d_*_·*sw*_). On the other hand, during the stance phase, the leg is extended to a target length (*l_d_*_·*st*_) and swung backward to a target angle (*θ_d_*_·*st*_) to propel the model. Meanwhile, the extended leg is kept at the constant target length (*l_d_*_·*st*_) by the PD control, and therefore, the linear leg works as a virtual spring where *l_d_*_·*st*_ is its natural length, such as in the SLIP model. The two simple motions alternate according to the CPG output in each leg. The equation of the PD controller of each shoulder (hip) joint used is shown in Section 2 of Supplementary Methods 1.

### Afferent feedback to the CPG on each leg

Andersson et al.^69^ reported that a sinusoidal hip movement entrains the rhythm of the CPG during fictive locomotion, and that feedback from the hip joint can exert the central network in generating fictive locomotion. Many modeling studies have demonstrated that it is very important to adjust the CPG through this hip (or shoulder) joint feedback if successful steady locomotion is to be achieved^15^,^24^,^49^,^64^. For our model, each hip joint's angle was multiplied by a constant gain, *k*_1_ in Fig. 7(a), and simply inputted into the half-centers of each leg. The equations are shown in Section 3 of Supplementary Methods 1. Without this feedback, the model would gradually drift away from the CPG rhythm during stepping, and not have been able to walk. Similar joint feedback mechanisms have been utilized by other modeling studies that employed Matsuoka's CPG model^24^,^26^,^49^,^63^,^64^.

We newly employ the leg loading negative feedback to the flexor half-center shown in Fig. 1 for postural adaptation. Specifically, the sensory feedback can be defined as
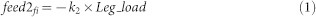
where *k*_2_ and *Leg_load* represent the weight and leg load (N) detected by a tactile sensor on each foot, respectively. *feed*2*_fi_* is inputted into the flexor half-center of the *i*th leg, as shown in Fig. 7(a), through Eq. (4) in Supplementary Methods 1. *k*_2_ = 0 denotes the model without the leg loading feedback described in the “Results” section.

The values of *k*_1_ and *k*_2_ are the same among the four legs.

## Author Contributions

Y.H. and T.F. performed the simulations. Y.F. analysed data and wrote the manuscript.

## Supplementary Material

Supplementary Information Gait generation.

Supplementary Information Gait transition.

Supplementary Information Supplementary Method
