# Supplementary material for: A simple rule for quadrupedal gait generation determined by leg loading feedback: a modeling study
Source: Sci Rep. 2015 Feb 2;5:8169. doi: 10.1038/srep08169 (PMC4313093; doi:10.1038/srep08169)
Supplement: Supplementary Information — Supplementary Method [file srep08169-s3.pdf]

Supplementary Method
# of the paper entitled "A simple rule for quadrupedal gait generation determined by leg loading feedback: a modeling study"
by Yasuhiro Fukuoka, Yasushi Habu and Takahiro Fukui

1 Half-center CPG model on each leg and the CPG's network

The following set of differential equations governs the CPG model for each leg.

$$
\begin{aligned}
T_r\dot{u}_{ei} + u_{ei} &= -\sum_{j=1}^{4}(p_{ij}[u_{ej}]^+ + q_{ij}[u_{fj}]^+) + s \\
&\quad -bv_{ei} + feed1_{ei}, &(1)\\
T_a\dot{v}_{ei} + v_{ei} &= y_{ei}, &(2)\\
y_{ei} &= [u_{ei}]^+ = \max\,(u_{ei}, 0), &(3)\\
T_r\dot{u}_{fi} + u_{fi} &= -\sum_{j=1}^{4}(p_{ij}[u_{fj}]^+ + q_{ij}[u_{ej}]^+) + s \\
&\quad -bv_{fi} + feed1_{fi} + feed2_{fi}, &(4)\\
T_a\dot{v}_{fi} + v_{fi} &= y_{fi}, &(5)\\
y_{fi} &= [u_{fi}]^+ = \max\,(u_{fi}, 0), &(6)
\end{aligned}
$$

$$
P_{i,j} = \begin{bmatrix} 0 & \beta & \alpha & 0 \\ \beta & 0 & 0 & \alpha \\ \alpha & 0 & 0 & \beta \\ 0 & \alpha & \beta & 0 \end{bmatrix}, Q_{i,j} = \begin{bmatrix} \gamma & 0 & 0 & 0 \\ 0 & \gamma & 0 & 0 \\ 0 & 0 & \gamma & 0 \\ 0 & 0 & 0 & \gamma \end{bmatrix} \qquad (7)
$$

where the suffix e, f, and i denotes the extensor, the flexor, and the number of leg (i.e., 1: left fore; 2: left hind; 3: right fore; 4: right hind), respectively. Eqs. (1)-(3) and (4)-(6) show the extensor and flexor activities. u_{ei} and u_{fi} indicate an inner state modeled on a membrane potential of the extensor and flexor neurons of the ith leg. v_{ei} and v_{fi} indicate the variables that represent the degrees of fatigue in the extensor and flexor neurons of the ith leg. The basic frequency of the CPG is determined by T_r and T_a, which are time constants of $u_{\{e,f\}i}$ and $v_{\{e,f\}i}$. s is a constant modeled on the descending signal from the brainstem. b is a constant for the recurrent inhibition of the inner state. The inhibitory synaptic connections between neurons as shown in Fig. 7(a) are represented by $\sum_{j=1}^{4}(p_{ij}[u_{\{e,f\}j}]^+ + q_{ij}[u_{\{f,e\}j}]^+)$, and the constants p_{ij} and q_{ij} are shown as Eq. (7), where the weights α and β determine the interlimb coordination and represent the connection strengths between the contralateral neurons and ipsilateral neurons, respectively, and the extensor and flexor half-centers on each leg mutually inhibit one another through the weight γ. $feed1_{\{e,f\}i}$ and $feed2_{fi}$ represent the sensory feedbacks (e.g., $feed1_{ei}$ is a feedback to the extensor neuron of the ith leg). y_{ei} and y_{fi} are the outputs of the extensor and flexor neurons of the ith leg, which are discontinuous terms via Eqs. (3) and (6). All of the parameter values of each CPG are the same for the four legs.

2 PD controllers on each leg

Specifically, the equation of the PD controller of each shoulder (hip) joint used can be shown as:

$$
\tau_i = K^{\tau}_{pi\cdot\{sw,st\}}(\theta_{di\cdot\{sw,st\}} - \theta_i) - K^{\tau}_{vi\cdot\{sw,st\}}\dot{\theta}_i, \qquad (8)
$$

where τ_i, θ_i and $\dot{\theta}_i$ are the output torque, the present angle, and the present angular velocity of the shoulder (hip) joint of the ith leg in Fig. 7(b), respectively. $\theta_{di\cdot\{sw,st\}}$ are the target shoulder (hip) joint angle during the swing/stance phase of the ith leg in Fig. 7(a). $K^{\tau}_{pi\cdot\{sw,st\}}$ and $K^{\tau}_{vi\cdot\{sw,st\}}$ are the proportional gain (P-gain) and the derivative gain (D-gain) in the swing/stance phase of the ith leg, respectively. The PD control of each linear elbow (knee) joint can be shown as:

$$
F_i = K^{F}_{pi\cdot\{sw,st\}}(l_{di\cdot\{sw,st\}} - l_i) - K^{F}_{vi\cdot\{sw,st\}}\dot{l}_i, \qquad (9)
$$

where F_i, l_i and $\dot{l}_i$ are the output force, the present length, and the present velocity of the linear elbow (knee) joint of the ith leg in Fig. 7(b), respectively; $l_{di\cdot\{sw,st\}}$ represents the target length of the linear elbow (knee) joint in the swing/stance phase of the ith leg in Fig. 7(a). $K^{F}_{pi\cdot\{sw,st\}}$ and $K^{F}_{vi\cdot\{sw,st\}}$ indicate P and D gains in the swing/stance phase of the ith leg. $K^{F}_{pi\cdot st}$ corresponds to the spring constant of the SLIP model-like linear spring in the stance phase.

3 Afferent feedback to the CPG on each leg

The hip joint feedback used for our model can be shown as:

$$\begin{aligned} feed1_{ei} &= k_1(\theta_i - \theta_0), \text{ and} \\ feed1_{fi} &= -k_1(\theta_i - \theta_0) \end{aligned} \tag{10}$$

where k_1, θ_i and θ_0 are the weight, the present shoulder (hip) joint angle of the ith leg, and the origin of each shoulder (hip) joint angle, respectively, as shown in Fig. 7(b). By putting these into Eqs. (1) and (4), we allow each leg to swing steadily across the center of θ_0.

The leg loading feedback is detailed in the main text.

4 Speed parameters for speed control

The values of the parameters used in all of the equations (Eqs. (1)-(10), and (1) in the main text) are constant at a constant speed, and were empirically determined so that the quadruped model could locomote safely at each constant speed. For example, the values of the parameters used in the simulation during which the quadruped model was trotting at a speed of 1.1 m/s in Fig. 3(c) are shown in Table 1.

To change its speed, we adjusted the values of the speed parameters (s, T_r, $\theta_{d \cdot st}$, $K^{\tau}_{pi \cdot st}$, $K^{F}_{pi \cdot st}$, $l_{d \cdot sw}$ and $K^{F}_{pi \cdot sw}$) that independently governed each leg's trajectory and walking cyclic duration, while all other values were constant irrespective of changes in the speed. Specifically, we implemented the following changes to increase the speed:

(a) Based on the physiological findings, we increased the tonic descending input from the brainstem to every half-center, s in Eqs. (1) and (4).

(b) To shorten the cyclic duration, we decreased the parameters that governed the cycle of each half-center, T_r in Eqs. (1) and (4).

(c) To swing each leg further backwards in the stance phase for propulsion, we decreased the target shoulder (hip) joint angle in the stance phase, $\theta_{d \cdot st}$ in Fig. 7(a) and Eq. (8), and to quickly reach it, we increased the control gain, $K^{\tau}_{pi \cdot st}$ in Eq. (8).

(d) To increase the stiffness of each support leg according to the speed, we increased the control gain, $K^{F}_{pi \cdot st}$ in Eq. (9), which corresponded to the modulus of the elasticity of the linear elbow (knee) joint.

(e) To avoid tripping during the swing phase, we decreased the target length of the linear elbow (knee) joint, $l_{d \cdot sw}$ in Fig. 7(a) and Eq. (9), and we increased its control gain, $K^{F}_{pi \cdot sw}$ to quickly shorten each leg.

It should be noted that these parameters in (a)-(e) exist to independently control each leg's oscillation, that the CPGs are always hard-wired (i.e., the connection weights α and β between the CPGs in Fig. 7(a) that directly dominated the phase relation among legs are always fixed) even during the changes of speed, and that the feedback gains to each leg's CPG, k_1 in Eq. (10) and k_2 in Eq. (1) in the main text, are also the same among the four legs.

Table 1: Values of the parameters used in a simulation where the quadruped model was trotting at 1.1 m/s in Fig. 3(c).

Parameter	Value	Parameter	Value
T_r	0.0473	$\theta_{di \cdot st}$	-0.7 rad
T_a	0.6	$K^{\tau}_{pi \cdot sw}$	7.6 Nm/rad
s	1.71	$K^{\tau}_{vi \cdot sw}$	1.0 Nms/rad
b	3.0	$K^{\tau}_{pi \cdot st}$	8.13 Nm/rad
α	-0.3	$K^{\tau}_{vi \cdot st}$	1.0 Nms/rad
β	-0.8	$l_{di \cdot sw}$	57 mm
γ	-2.0	$l_{di \cdot st}$	130 mm
θ_0	-0.262 rad	$K^{F}_{pi \cdot sw}$	1.86 kN/m
k_1	3.0	$K^{F}_{vi \cdot sw}$	40.0 Ns/m
k_2	0.08	$K^{F}_{pi \cdot st}$	1.97 kN/m
$\theta_{di \cdot sw}$	1.05 rad	$K^{F}_{vi \cdot st}$	40.0 Ns/m
